# Molecular Structure and Internal Dynamics of 2^′^-Hydroxyacetophenone by Free-Jet Absorption Millimeter-Wave Spectroscopy

**DOI:** 10.3390/molecules29245842

**Published:** 2024-12-11

**Authors:** Salvatore Boi, Sonia Melandri, Luca Evangelisti, Assimo Maris

**Affiliations:** 1 Department of Chemistry G. Ciamician, University of Bologna, 40126 Bologna, Italy; salvatore.boi@studio.unibo.it (S.B.);; 2 Interdepartmental Centre for Industrial Aerospace Research (CIRI Aerospace), University of Bologna, 47521 Cesena, Italy; 3 Interdepartmental Centre for Industrial Agrifood Research (CIRI Agrifood), University of Bologna, 47121 Forlì, Italy; 4 Department of Chemistry G. Ciamician, U.O.S. Ravenna, University of Bologna, 48123 Ravenna, Italy

**Keywords:** rotational spectroscopy, methyl internal rotation, molecular structure, aromatic molecules, supersonic expansion

## Abstract

The rotational spectrum of $2^{\prime }$-hydroxyacetophenone has been recorded and assigned for the first time using a Stark-modulated free-jet absorption millimeter-wave (FJ-AMMW) spectrometer in the 59.6–74.5 GHz frequency range. The most stable conformer has been detected and assigned: *A* = 2277.076(11), *B* = 1212.113(5) and *C* = 795.278(5) MHz. It is characterized by a $C_{s}$ symmetry where a strong hydrogen bond between the acetyl oxygen atom and the hydroxyl atom takes place. The transition lines show a fine structure due to the internal rotation of the methyl group, which allowed the determination of a $V_{3}$ = 565.1(5) ${\mathrm{cm}}^{-1}$ barrier. The corresponding tunneling splittings have been estimated to be 51 MHz. Calculations at the B3LYP-D3(BJ)/Def2-TZVP level underestimate the height of the barrier by about 156 ${\mathrm{cm}}^{-1}$. This value decreases to 25 ${\mathrm{cm}}^{-1}$ with MP2/aug-cc-pVTZ.

## 1. Introduction

Rotational spectroscopy is a tool suited to obtain information on the structure of molecules and weakly bound molecular complexes in the gas phase, with the rotational constants being directly related to the atomic masses’ distribution in space. An example is provided by the study of the 1:1 acrolein–water system for which four systems were identified corresponding to the s-*trans* conformer of acrolein forming a cycle with one or two molecules of water through the HC=O or the HCC=O frames [1]. Moreover, in the same study, the behavior of the ^18^O isotopologues proved the presence of an oxygen exchange process between acrolein and water.

Besides the rigid molecular structure, the rotational spectra encode the effects of the vibrational modes. In particular, large amplitude motions leading to equivalent minima such as the inversion motion, the internal rotation, the ring-puckering and the pseudo-rotation produce typical splitting of the rotational transition lines that can be disentangled to obtain precise information on the underlying potential energy surface (PES). Among them, methyl internal rotation has been long studied [2]. There are experimentally determined barriers hindering a methyl torsion range from few ${\mathrm{cm}}^{-1}$ (i.e., 2-butynoic acid $V_{3}$ = 1.0090(4) ${\mathrm{cm}}^{-1}$ [3]) to more than 1000 ${\mathrm{cm}}^{-1}$ (i.e., ethyl vinyl ether $V_{3}$ = 1074.4(4) ${\mathrm{cm}}^{-1}$ [4,5], methylcyclopropane $V_{3}$ = 1001(17) ${\mathrm{cm}}^{-1}$ [6], 1-chloro-1-fluoroethane $V_{3}$ = 1334(4) ${\mathrm{cm}}^{-1}$ [7]).

Intermediate barriers between the low- and high-energy limits have been observed for methyl groups in acyl compounds (${R-CO-CH}_{3}$), the exact value being determined by the nature of the whole molecule. The barrier in the prototype system acetaldehyde (R = H) is $V_{3}$ = 400(2) ${\mathrm{cm}}^{-1}$ [8]. Alkyl substitution typically lowers this value, for instance in acetone (R = Me) $V_{3}$ = 266.16(5) ${\mathrm{cm}}^{-1}$ [9] and in methyl ethyl ketone (R = Et) $V_{3}$ = 181.5(1) ${\mathrm{cm}}^{-1}$ [10]. The lowering effect seems more pronounced in the case of alkoxy substituents, as seen for instance for methyl acetate (R = MeO) $V_{3}$ = 99.56(8) ${\mathrm{cm}}^{-1}$ [11], ethyl acetate (R = EtO) $V_{3}$ = 99.6(1) ${\mathrm{cm}}^{-1}$ [12], vinyl acetate (R = CH_2_CHO) $V_{3}$ = 155.10(8) ${\mathrm{cm}}^{-1}$ [13] and phenyl acetate (R = PhO) $V_{3}≃$ 143 ${\mathrm{cm}}^{-1}$ [14].

Interestingly, in the cases of pyruvic acid (R = COOH) and methylpyruvate (R = COOMe) the barriers are closer to the values for acetaldehyde, $V_{3}$ = 349(18) [15] and 389.4(7) [16] ${\mathrm{cm}}^{-1}$, respectively. Similar values are also found in α- and β-ionone (R = CH=CH-CH-trimethylcyclo-hexene) [17]. In particular, the barriers in conformers showing an s-*cis* arrangement of the C=C-C=O frame are slightly lower than the value of acetaldehyde ($V_{3}\left(\alpha \right)≃$ 360 and $V_{3}\left(\beta \right)≃$ 340 ${\mathrm{cm}}^{-1}$), whereas the barriers in s-*trans* forms are slightly higher ($V_{3}\left(\alpha \right)≃$ 445 and $V_{3}\left(\beta \right)≃$ 430 ${\mathrm{cm}}^{-1}$) [17].

Phenyl substitution (R = Ph) has an opposite effect with respect to alkyl substitution; the methyl internal rotation barrier increases up to $V_{3}$ = 627(3) ${\mathrm{cm}}^{-1}$ in acetophenone [18]. Focusing on acetophenone derivatives, recent studies show that a single substitution on the aromatic ring slightly decreases the barrier: $V_{3}$ = 606(1) ${\mathrm{cm}}^{-1}$ in $4^{\prime }$-fluoroacetophenone [19], $V_{3}≃$ 588(3) ${\mathrm{cm}}^{-1}$ in $4^{\prime }$-methylacetophenone [20], $V_{3}$ = 545.7(5) ${\mathrm{cm}}^{-1}$ in $4^{\prime }$-aminoacetophe-none [21] and $V_{3}$ = 594(1) ${\mathrm{cm}}^{-1}$ in $3^{\prime }$-aminoacetophenone [21]. Double substitution in hydroxy-methoxyacetophenones has a similar effect, and, notably, also the orientation of the methoxy group affects the height of the barrier: $V_{3}$ = 588(4)/612(3) ${\mathrm{cm}}^{-1}$ in a/s-*trans* 6-hydroxy-3-methoxyacetophenone and $V_{3}$ = 552(2)/622(10) ${\mathrm{cm}}^{-1}$ in s-*cis*/*trans* 4-hydroxy-3-methoxyacetophenone, respectively [22]. However, an opposite trend has been recently enlightened in $2^{\prime }$-aminoacetophenone, for which the determined barrier is $V_{3}$ = 644(3) ${\mathrm{cm}}^{-1}$ [23].

**Figure 1:**
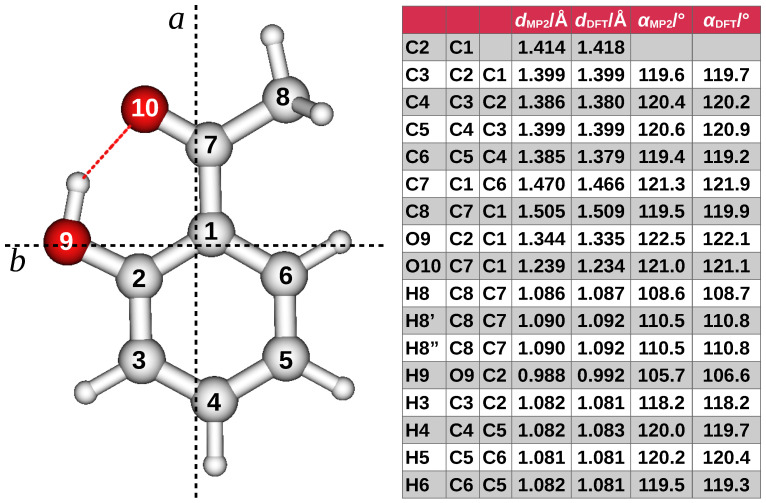
(**Left**) ball-and-stick model, numbering, and principal axis system of 2HA. Red is used for oxygen and white for carbon and hydrogen (smaller size). The rotatable bonds are C1-C7, C2-O9 and C7-C8. (**Right**) theoretical internal coordinates (distances and angles) at the B3LYP-D3(BJ)/Def2-TZVP (DFT) and MP2/aug-cc-pVTZ levels of calculation. All the atoms lie in the symmetry plane except the out-of-plane methyl hydrogen atoms. The dihedral angles formed with the in-plane methyl hydrogen atom are HC8C7H = ±120.4° at both levels.

To further explore the effect of the substituents on the methyl internal rotation barrier of acetophenone, here we present a rotational spectroscopy study of $2^{\prime }$-hydroxyaceto-phenone (2HA, Figure 1), also known as 2-acetylphenol, performed using free-jet absorption millimeter-wave (FJ-AMMW) spectroscopy.

## 2. Results

**Figure 2:**
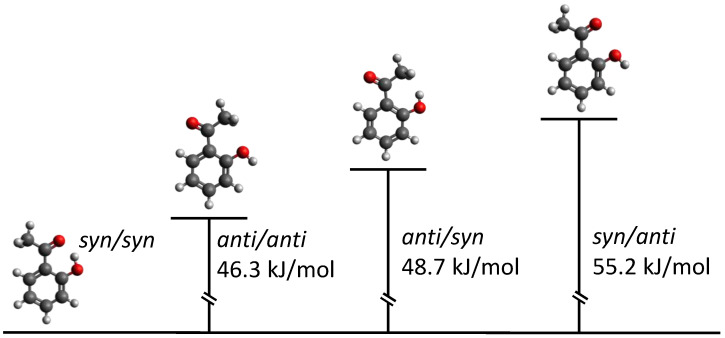
Theoretical relative energy diagram ($\Delta E_{e}$) of the four 2HA conformers obtained at the B3LYP-D3(BJ)/Def2-TZVP level of calculation ($E_{e}^{syn/syn}$ = −460.345522 a.u.). Red is used for oxygen, grey for carbon and white hydrogen.

A preliminary DFT scan B3LYP-D3(BJ)/Def2-TZVP of the torsional dihedral angles shows that four conformers exist, according to the *syn* or *anti* orientation of the carbonyl and hydroxyl groups with respect to the C-C phenyl bond connecting the groups themselves. They are shown in Figure 2 with the relative energy values. All conformers have a $C_{s}$ symmetry with all the atoms lying on the plane of symmetry except the methyl hydrogen ones. The global minimum (*syn*/*syn*) is characterized by the hydrogen bond between the hydroxyl and the carbonyl group. The remaining conformers lie more than 45 kJ ${\mathrm{mol}}^{-1}$ above the global minimum. Among them, the more stable is the *anti*/*anti* species where the methyl group faces the hydroxyl oxygen atom and the carbonyl is toward the vicinal phenyl hydrogen atom. However, due to the huge energy difference, their abundance at the experimental conditions is expected to be negligible. As regards the global minimum, the theoretical results were refined by means of ab initio calculations performed at the MP2/aug-cc-pVTZ level. The DFT and ab initio internal coordinates are compared in the right side of Figure 1. The bond distances and angles differ for less than 1 nm and 1°, respectively. However, the largest discrepancies are observed for the hydroxyl group and the carbon atom of the acetyl frame. The Gaussian input and output files for the four conformers of 2HA and the corresponding geometries in Cartesian coordinates can be found in the AMS Acta repository [24].

**Figure 3:**
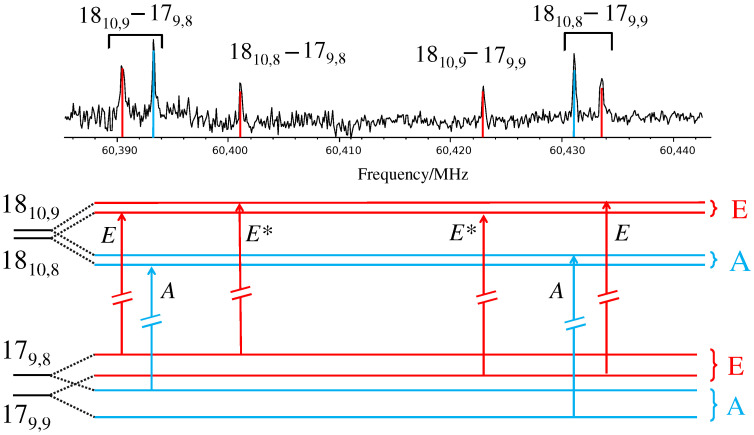
Portion of the recorded spectrum of 2HA showing the $18_{10,x}\leftarrow 17_{9,y}$ transition lines and scheme of the involved levels. The most intense peaks correspond to the $\mu_{b}$ A-symmetry transitions; the outer peaks are the $\mu_{b}$ E-symmetry transitions while the inner ones are the forbidden $\mu_{c}$ E-symmetry lines (labeled with an asterisk). The colored sticks in the upper part evidence the frequency of the transitions; their height does not represent the predicted intensity.

The spectroscopic parameters predicted for the global minimum are listed in Table 1. The DFT and ab initio calculations estimate the $\mu_{b}$ electric dipole moment component to be about 3 D. Accordingly, several $R-\mu_{b}$-type transition lines were detected with $J^{\prime \prime }$ values ranging from $J^{\prime \prime }$ = 14 to $J^{\prime \prime }$ = 30 and $K_{a}^{\prime \prime }$ values from $K_{a}^{\prime \prime }$ = 7 to $K_{a}^{\prime \prime }$ = 16. The observed lines show a fine structure due to methyl internal rotation: each transition line splits into the A and E components, involving the non-degenerate and the double-degenerate levels, respectively. Moreover, several E-symmetry transition lines with low $K_{c}$ show additional components because of the presence of the “electric dipole forbidden transitions”, which apparently follow $\mu_{c}$-type selection rules [25]. As an example, the portion of the spectrum recorded in the 60,385-60,445 MHz frequency region is given in Figure 3 where the fine structure of the $18_{10}-17_{9}$ transition lines and the scheme of the involved levels are shown.

The overall set of detected lines was analyzed using the combined axis method (CAM) [26] implemented in the XIAM program [27], which fits a set of spectroscopic constants common to both the A and E states and directly supplies the methyl internal rotation barrier. The Hamiltonian for a one-top problem can be written as:

(1) $$H=H_{R}+H_{CD}+D^{-1}\times H_{i}\times D$$

where unique rigid rotor $H_{R}$ and centrifugal distortion $H_{CD}$ operators are treated in the principal axis system (PAS) and used for both the A and E-states, while the internal rotation Hamiltonian $H_{i}$ is set up in the rho-axis system (RAM) and then rotated into the PAS using a rotation matrix *D*. As regards the internal rotation parameters, the barrier of a three-fold potential ($V_{3}$) was freely optimized during the fitting procedure while the values of geometrical parameters such as the reduced internal rotation constant ($F_{0}$) and the angles between the internal rotor axis (*i*) and the principal axes of inertia were fixed to those of the calculated structures. In particular, assuming that the methyl internal rotation axis (*i*) matches the C7-C8 bond, the (i,c) angle was fixed to $90^{\circ }$, while the (i,a) angle was fixed to the value of the angle between the C7-C8 bond and the *a* axis. The complete line list is reported in Table 2 while the spectroscopic constants obtained using the *S*-reduction in $I^{r}$-representation are given in Table 1.

## 3. Discussion

**Figure 4:**
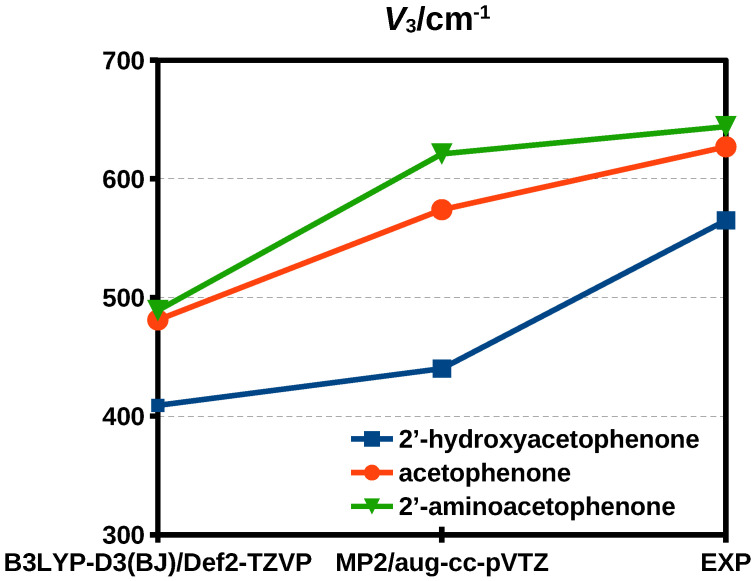
Comparison of the theoretical and experimental methyl internal rotation barrier values of $2^{\prime }$-hydroxyacetophenone, acetophenone and $2^{\prime }$-aminoacetophenone.

Taking into account that calculations describe the equilibrium geometry ($r_{e}$) while observations are related to the vibrational ground state ($r_{0}$), the agreement between the theoretical and experimental rotational constants is fair. Indeed, both DFT and ab initio overestimate the observed values by less than 1%. The values of the planar moments of inertia ($M_{gg}=\Sigma_{i}m_{i}g_{i}^{2}$, *g* = *a*, *b* or *c*) listed in Table 1 show that the distribution of the masses along the principal axes is more spread for the observed than the calculated ones. In particular, the small value of $M_{cc,0}$ = 1.704(3) ${\mathrm{uÅ}}^{2}$ confirms that 2HA has a $C_{s}$ symmetry, where all the atoms lie in the ab inertial plane except the methyl hydrogen atoms. The discrepancy between the observed and theoretical values ($M_{cc,0}-M_{cc,e}$ = 0.142 ${\mathrm{uÅ}}^{2}$) suggests the presence of an out-of-plane large amplitude motion [28]. Previous studies on the parent system acetophenone show that it is characterized by a $C_{s}$ geometry [18,29] and that its $M_{cc,0}$ = 1.79461(7) ${\mathrm{uÅ}}^{2}$ is affected by the contribution of the acetyl torsion, whose fundamental wavenumber determined by far infrared spectroscopy is quite small ${\tilde{v}}_{01}$ = 49.5 ${\mathrm{cm}}^{-1}$ [30]. As concerns the hydroxyl group, its motion in phenol is characterized by a two-fold PES that leads to an $M_{cc,0}$ = 0.014767(1) ${\mathrm{uÅ}}^{2}$ value [31,32]. It is worth noting that the $M_{cc,0}$ of 2HA is smaller than that of acetophenone, in agreement with a stiffening of the acetyl and hydroxyl torsions due to the hydrogen bond between them. The same effect has been found in the case of $2^{\prime }$-aminoacetophenone, where the hydrogen bond takes place between the acetyl and the amino group [33]. As regards the methyl internal rotation potential energy barrier, a threefold path with a barrier of $V_{3}$ = 565.1(4) ${\mathrm{cm}}^{-1}$ has been found. Based on this potential energy model, the splitting between the A and E levels for the vibrational ground state can be determined as [2]:

(2) $$\Delta_{0}=E_{E}-E_{A}=\frac{27}{8}\cdot F\cdot w_{1}^{v=0}$$

where *F* is the reduced rotational constant for internal rotation and $w_{1}^{v=0}$ is the Fourier coefficient of Mathieu eigenvalues for the vibrational ground state associated with the reduced barrier *s* [34]. For 2HA, using *F* = 161,740 MHz, *s* = 46.12 and $w_{1}^{v=0}$ = 9.42 × $10^{-5}$, the estimated tunneling splitting is $\Delta_{0}$ = 51 MHz. In Figure 4, the experimental and theoretical barrier values of 2HA are compared to those of acetophenone and $2^{\prime }$-aminoacetophenone. MP2/aug-cc-pVTZ performs better than B3LYP-D3(BJ)/Def2-TZVP in estimating the absolute values of the methyl internal rotation barriers, but the variation of the barrier when comparing different compounds seems better predicted with the DFT approach.

## 4. Experimental Methods

A sample of 2HA (C_8_H_8_O_2_, CAS no. 18-93-4, InChIKey JECYUBVRTQDVAT-UHFFF-AOYSA-N) was purchased from Thermo Fisher Scientific (Waltham, MA, USA) (purity 99%) and used without further purification. It was analyzed in the 59.6–74.4 GHz frequency region using a Stark-modulated free-jet absorption millimeter-wave (FJ-AMMW) spectrometer, which was previously described [35,36]. A 3000 V Stark voltage was applied to two aluminum plates placed at a distance of 4 cm with a 33 kHz repetition rate. The estimated accuracy of the frequency measurements is about 50 kHz, allowing the resolution of lines separated by >300 kHz. The sample (b.p. 479 K, m.p. 276–279 K) was loaded into a sample reservoir, inserted into an argon line ($P_{0}$ = 21 kPa) and heated to *T* = 343 K to obtain sufficient vapor pressure. The gas mixture was expanded into a vacuum chamber through a 0.3 mm pinhole nozzle (with a final pressure of about $P_{b}$ = 0.5 Pa), allowing for the recording of the rotational spectrum under supersonic jet expansion conditions, for which the estimated rotational temperature is about 10 K [37,38].

## 5. Computational Methods

Geometry optimizations and harmonic vibrational frequency calculations were performed with the Gaussian16^®^ software package (G16, Rev. A.03) (Gaussian is a registered trademark of Gaussian, Inc. 340 Quinnipiac St. Bldg. 40 Wallingford, CT 06492 USA.) using both the ab initio and density functional theory (DFT) approaches. As regards DFT, the Becke-three-parameters Lee–Yang–Parr hybrid functional (B3LYP [39,40]) was used corrected with the D3 version of Grimme’s dispersion with Becke–Johnson damping (D3(BJ) [41]) together with valence triple-ζ-quality Karlsruhe polarized type basis set (Def2-TZVP [42]). This approach has been proven to provide a good combination of accuracy and computational efficiency for the simulation of isolated organic molecules [43] and non-covalent interactions [44]. Selected ab initio calculations were then performed through the Møller–Plesset second-order perturbation theory (MP2 [45,46]) using triple-ζ-quality Dunning correlation consistent polarized type basis set augmented with diffuse functions (aug-cc-pVTZ [47]).

## 6. Conclusions

In this work, the rotational spectrum of 2HA has been recorded and assigned for the first time. A total of 134 $\mu_{b}$-type transition lines have been observed. These lines show a fine structure related to the methyl internal rotation tunneling, which splits each transition into A and E components. A global fit performed with XIAM, considering both A and E lines, whether forbidden or permitted, allowed the determination of the three rotational constants, all quartic centrifugal distortion constants, and the methyl internal rotation barrier. Comparison with simulated data probed that MP2/aug-cc-pVTZ calculations are better than the B3LYP-B3(DJ)/Def2-TZVP ones in reproducing the absolute $V_{3}$ values, whereas the latter shows a better trend of the variation in the methyl internal rotation barrier of 2HP and $2^{\prime }$-aminoacetophenone to the parent analogous acetophenone.

## Figures and Tables

**Table 1:** Experimental and theoretical spectroscopic parameters of the most stable conformation of $2^{\prime }$-hydroxyacetophenone in the *S*-reduction and $I^{r}$ representation.

${\mathrm{Parameter}\;}^{a}$	${\mathrm{DFT}\;}^{b}$	EXP. ${\mathrm{(XIAM)}\;}^{c}$	${\mathrm{MP2}\;}^{d}$	EXP. ${\mathrm{(XIAM)}\;}^{e}$
*A*/MHz	2292.530	2277.076(10) ^f^	2278.667	2277.076(10)
*B*/MHz	1219.819	1212.1125(40)	1220.725	1212.1122(42)
*C*/MHz	800.120	795.2771(44)	798.814	795.2772(46)
$D_{J}$/Hz	23.9	25.9(29)	24.0	25.9(30)
$D_{JK}$/Hz	25.2	28.2(42)	26.7	28.2(44)
$D_{K}$/Hz	115.0	118.1(56)	118.1	118.1(59)
$d_{1}$/Hz	−9.6	−5.3(20)	−9.7	−5.3(20)
$d_{2}$/Hz	−1.9	−4.6(14)	−1.9	−4.6(15)
$V_{3}$/${\mathrm{cm}}^{-1}$	409	565.5(4)	540	565.1(4)
*N*	-	134	-	134
σ/MHz	-	0.064	-	0.068
$M_{aa}$/${\mathrm{uÅ}}^{2}$	412.745	[415.237(3)] ^g^	412.437	[415.237(3)]
$M_{bb}$/${\mathrm{uÅ}}^{2}$	218.426	[220.238(3)]	220.225	[220.238(3)]
$M_{cc}$/${\mathrm{uÅ}}^{2}$	1.562	[1.704(3)]	1.562	[1.704(3)]
$\mu_{a}$/D	0.94	-	0.77	-
$\mu_{b}$/D	3.13	-	3.15	-
κ	−0.438	[−0.437]	−0.430	[−0.437]
$\Delta_{0}$/MHz	303	[50]	68	[51]

^a^ *A*, *B* and *C* are the rotational constants. $D_{J}$, $D_{JK}$, $D_{K}$, $d_{1}$ and $d_{2}$ are the quartic centrifugal distortion constants. $V_{3}$ is the methyl internal rotation barrier. *N* is the number of transition lines fitted. σ is the standard deviation of the fit. κ=(2B−A−C)/(A−C) is the Ray’s asymmetry parameter. $M_{gg}\;=\;\Sigma_{i}m_{i}g_{i}^{2}$ (*g* = *a*, *b* or *c*) are the planar moments of inertia. $\mu_{g}$ (*g* = *a*, *b* or *c*) are the electric dipole moment components, $\mu_{c}$ is null by symmetry. $\Delta_{0}\;=\;E_{E}-E_{A}$ is the methyl internal rotation tunneling splitting. ^b^ B3LYP-D3(BJ)/Def2-TZVP. ^c^ Structural parameters in the fit are fixed to the B3LYP-D3(BJ)/Def2-TZVP values: $F_{0}$ = 159.58 GHz, $\hat{i,a}=56^{\circ }$, $\hat{i,c}=90^{\circ }$ (see text for details). ^d^ MP2/aug-cc-pVTZ. ^e^ Structural parameters in the fit are fixed to the MP2/aug-cc-pVTZ values: $F_{0}$ = 161.74 GHz, $\hat{i,a}=59^{\circ }$, $\hat{i,c}=90^{\circ }$ (see text for details). ^f^ Error in the unit of the last digit. ^g^ Values in squared brackets are derived from the fitted parameters.

**Table 2:** Measured frequencies (ν/MHz) and fitted deviations (c−o/MHz) of the rotational A- and E-symmetry transition lines of 2-hydroxyacetophenone. Transitions are labeled according to the quantum numbers of the involved levels: $J"\left(K_{a}",K_{c}"\right)-J^{\prime }\left(K_{a}^{\prime },K_{c}^{\prime }\right)$.

	$\nu_{A}$	c−o		$\nu_{E}$	c−o		$\nu_{A}$	c−o		$\nu_{E}$	c−o
14(13,1)-13(12,2)	59,987.54	−0.01	14(13,2)-13(12,2)	59,986.92	−0.02	19(9,10)-18(8,11)	60,030.62	−0.06	19(9,10)-18(8,11)	60,030.62	0.04
14(13,2)-13(12,1)	59,987.54	−0.01	14(13,1)-13(12,1)	59,987.79	−0.04	19(10,9)-18(9,10)	62,405.54	−0.02	19(10,9)-18(9,10)	62,406.51	0.05
14(14,1)-13(13,0)	62,500.40	0.16	14(14,1)-13(13,1)	62,499.65	0.02	19(10,10)-18(9,9)	62,298.94	−0.05	19(10,10)-18(9,9)	62,297.77	−0.01
14(14,0)-13(13,1)	62,500.40	0.16	14(14,0)-13(13,0)	62,500.40	−0.12	19(11,9)-18(10,8)	65,010.24	0.02	19(11,9)-18(10,9)	65,014.29	−0.03
15(13,3)-14(12,2)	62,027.50	0.06	15(13,3)-14(12,3)	62,026.82	−0.02	19(11,8)-18(10,9)	65,017.71	0.04	19(11,8)-18(10,8)	65,013.26	0.02
15(13,2)-14(12,3)	62,027.50	0.06	15(13,2)-14(12,2)	62,027.69	−0.04	19(12,8)-18(11,7)	67,601.49	0.07	19(12,8)-18(11,8)	67,600.99	0.00
15(14,2)-14(13,1)	64,541.52	0.09	15(14,2)-14(13,2)	64,540.78	−0.04	19(12,7)-18(11,8)	67,601.84	0.05	19(12,7)-18(11,7)	67,601.84	−0.04
15(14,1)-14(13,2)	64,541.52	0.09	15(14,1)-14(13,1)	64,541.66	−0.04	19(13,7)-18(12,6)	70,153.84	0.01	19(13,7)-18(12,7)	70,153.20	−0.01
15(15,1)-14(14,0)	67,053.98	−0.01	15(15,1)-14(14,1)	67,053.32	−0.05	19(13,6)-18(12,7)	70,153.84	0.00	19(13,6)-18(12,6)	70,154.07	−0.03
15(15,0)-14(14,1)	67,053.98	−0.01	15(15,0)-14(14,0)	67,054.20	−0.06	19(14,6)-18(13,5)	72,687.49	0.03	19(14,6)-18(13,6)	72,686.84	0.00
16(12,5)-15(11,4)	61,543.09	0.00	16(12,5)-15(11,5)	61,542.45	−0.04	19(14,5)-18(13,6)	72,687.49	0.03	19(14,5)-18(13,5)	72,687.70	−0.02
16(12,4)-15(11,5)	61,543.09	−0.01	16(12,4)-15(11,4)	61,543.30	−0.08	19(15,4)-18(14,5)	75,210.89	0.00	19(15,5)-18(14,5)	75,210.25	−0.01
16(13,4)-15(12,3)	64,065.17	−0.01	16(13,4)-15(12,4)	64,064.55	−0.02	19(15,5)-18(14,4)	75,210.89	0.00	19(15,4)-18(14,4)	75,211.07	−0.07
16(13,3)-15(12,4)	64,065.17	−0.01	16(13,3)-15(12,3)	64,065.40	−0.06	20(9,12)-19(8,11)	60,179.84	−0.06	20(9,12)-19(8,11)	60,179.84	0.05
16(14,3)-15(13,2)	66,581.60	0.02	16(14,3)-15(13,3)	66,580.95	−0.02	20(9,11)-19(8,12)	62,378.36	−0.06	20(9,11)-19(8,12)	62,378.36	0.11
16(14,2)-15(13,3)	66,581.60	0.02	16(14,2)-15(13,2)	66,581.81	−0.04	20(10,10)-19(9,11)	64,391.09	−0.02	20(10,10)-19(9,11)	64,391.29	−0.04
16(15,2)-15(14,1)	69,095.37	0.14	16(15,2)-15(14,2)	69,094.62	0.01	20(10,11)-19(9,10)	64,115.54	0.01	20(10,11)-19(9,10)	64,114.97	−0.03
16(15,1)-15(14,2)	69,095.37	0.14	16(15,1)-15(14,1)	69,095.37	−0.13	20(11,10)-19(10,9)	66,969.23	0.04	20(11,10)-19(10,9)	66,964.98	0.03
16(16,1)-15(15,0)	71,607.76	0.07	16(16,1)-15(15,1)	71,607.04	−0.02				20(11,10)-19(10,10)	66,985.80	0.02
16(16,0)-15(15,1)	71,607.76	0.07	16(16,0)-15(15,0)	71,607.89	−0.05				20(11,9)-19(10,9)	66,975.09	0.01
17(11,7)-16(10,6)	61,024.09	0.01	17(11,7)-16(10,7)	61,023.76	−0.01	20(11,9)-19(10,10)	66,992.01	0.01	20(11,9)-19(10,10)	66,995.95	0.04
17(11,6)-16(10,7)	61,024.62	−0.05	17(11,6)-16(10,6)	61,024.62	−0.04	20(12,9)-19(11,8)	69,599.75	0.07	20(12,9)-19(11,9)	69,599.75	−0.01
17(12,6)-16(11,5)	63,570.63	0.02	17(12,6)-16(11,6)	63,569.96	−0.05	20(12,8)-19(11,9)	69,601.09	0.06	20(12,8)-19(11,8)	69,600.59	−0.01
17(12,5)-16(11,6)	63,570.63	0.00	17(12,5)-16(11,5)	63,570.86	−0.04	20(13,8)-19(12,7)	72,170.55	0.19	20(13,8)-19(12,8)	72,169.76	0.00
17(13,5)-16(12,4)	66,099.69	0.01	17(13,5)-16(12,5)	66,099.05	−0.02	20(13,7)-19(12,8)	72,170.55	0.13	20(13,7)-19(12,7)	72,170.55	−0.09
17(13,4)-16(12,5)	66,099.69	0.01	17(13,4)-16(12,4)	66,099.90	−0.06	20(14,7)-19(13,6)	74,714.61	0.12	20(14,7)-19(13,7)	74,713.83	−0.03
17(14,4)-16(13,3)	68,619.93	0.01	17(14,4)-16(13,4)	68,619.27	−0.03	20(14,6)-19(13,7)	74,714.61	0.11	20(14,6)-19(13,6)	74,714.61	−0.13
17(14,3)-16(13,4)	68,619.93	0.01	17(14,3)-16(13,3)	68,620.15	−0.04	21(9,12)-20(8,13)	65,121.19	−0.16	21(9,12)-20(8,13)	65,121.19	0.08
17(15,3)-16(14,2)	71,135.61	0.02	17(15,3)-16(14,3)	71,134.96	0.00	21(9,13)-20(8,12)	60,881.07	−0.05	21(9,13)-20(8,12)	60,881.07	−0.01
17(15,2)-16(14,3)	71,135.61	0.02	17(15,2)-16(14,2)	71,135.76	−0.08	21(10,12)-20(9,11)	65,773.04	0.01	21(10,12)-20(9,11)	65,772.72	−0.03
17(16,2)-16(15,1)	73,649.03	0.07	17(16,2)-16(15,2)	73,648.30	−0.03	21(10,11)-20(9,12)	66,429.91	−0.04	21(10,11)-20(9,12)	66,429.91	0.00
17(16,1)-16(15,2)	73,649.03	0.07	17(16,1)-16(15,1)	73,649.03	−0.18	21(11,10)-20(10,11)	68,953.17	0.01	21(11,10)-20(10,11)	68,954.94	−0.02
18(10,9)-17(9,8)	60,393.27	0.01	18(10,9)-17(9,8)	60390.47	−0.01	21(11,11)-20(10,10)	68,888.82	−0.03	21(11,11)-20(10,10)	68,886.68	−0.02
			18(10,9)-17(9,9)	60,422.93	0.00	21(12,10)-20(11,9)	71,581.75	0.03	21(12,10)-20(11,10)	71,583.80	0.12
			18(10,8)-17(9,8)	60,401.12	0.00	21(12,9)-20(11,10)	71,586.18	−0.02	21(12,9)-20(11,9)	71,583.80	−0.08
18(10,8)-17(9,9)	60,431.10	0.01	18(10,8)-17(9,9)	60433.54	−0.03	21(13,9)-20(12,8)	74,177.54	0.23	21(13,9)-20(12,9)	74,176.81	0.01
18(11,8)-17(10,7)	63,025.86	−0.06	18(11,8)-17(10,8)	63,026.56	0.05	21(13,8)-20(12,9)	74,177.54	0.00	21(13,8)-20(12,8)	74,177.54	−0.14
18(11,7)-17(10,8)	63,028.19	0.06	18(11,7)-17(10,7)	63,027.28	0.06	22(9,14)-21(8,13)	61,051.48	0.07	22(9,14)-21(8,13)	61,051.48	0.06
18(12,7)-17(11,6)	65,590.85	0.06	18(12,7)-17(11,7)	65,590.20	−0.02	22(9,13)-21(8,14)	68,481.21	−0.14	22(9,13)-21(8,14)	68,481.21	0.18
18(12,6)-17(11,7)	65,590.85	−0.03	18(12,6)-17(11,6)	65,591.06	−0.05	22(10,13)-21(9,12)	67,159.40	−0.08	22(10,13)-21(9,12)	67,159.40	0.09
18(13,6)-17(12,5)	68,129.69	−0.02	18(13,6)-17(12,6)	68,129.06	−0.03	22(10,12)-21(9,13)	68,603.38	−0.08	22(10,12)-21(9,13)	68,603.38	0.06
18(13,5)-17(12,6)	68,129.69	−0.02	18(13,5)-17(12,5)	68,129.96	−0.02	23(9,15)-22(8,14)	60,774.22	0.04	23(9,15)-22(8,14)	60,774.22	−0.02
18(14,5)-17(13,4)	70,655.57	0.02	18(14,5)-17(13,5)	70,654.91	−0.02	23(10,14)-22(9,13)	68,130.46	−0.08	23(10,14)-22(9,13)	68,130.46	0.00
18(14,4)-17(13,5)	70,655.57	0.02	18(14,4)-17(13,4)	70,655.81	0.00	23(12,11)-22(11,12)	75,508.20	−0.05	23(12,11)-22(11,12)	75,511.45	0.05
18(15,4)-17(14,3)	73,174.49	0.10	18(15,4)-17(14,4)	73,173.72	−0.04	25(11,15)-24(10,14)	75,242.93	−0.14	25(11,15)-24(10,14)	75,242.93	0.01
18(15,3)-17(14,4)	73,174.49	0.10	18(15,3)-17(14,3)	73,174.49	−0.15	29(9,21)-28(8,20)	60,753.68	0.00	29(9,21)-28(8,20)	60,753.68	0.04
18(16,2)-17(15,3)	75,689.53	0.06	18(16,3)-17(15,3)	75,688.79	−0.05	30(7,23)-29(8,22)	59,727.16	−0.06	30(7,23)-29(8,22)	59,727.16	0.03
18(16,3)-17(15,2)	75,689.53	0.06	18(16,2)-17(15,2)	75,689.53	−0.18	30(8,22)-29(9,21)	61,223.83	−0.06	30(8,22)-29(9,21)	61,223.83	0.06

## Data Availability

The computational data supporting the findings of this study are openly available in the University of Bologna repository at https://doi.org/10.6092/unibo/amsacta/7984.
